# Supplementary material for: Prediction of Radiation-Induced Hypothyroidism Using Radiomic Data Analysis Does Not Show Superiority over Standard Normal Tissue Complication Models
Source: Cancers (Basel). 2021 Nov 8;13(21):5584. doi: 10.3390/cancers13215584 (PMC8582656; doi:10.3390/cancers13215584)
Supplement: Supplementary file 1 [file cancers-13-05584-s001.zip › cancers-1413372 Supplementary Figure after proof.pdf]

cancers

Supplementary Material: Prediction of Radiation-Induced Hypothyroidism Using Radiomic Data Analysis Does Not Show Superiority over Standard Normal Tissue Complication Models

Urszula Smyczynska, Szymon Grabia, Zuzanna Nowicka, Anna Papis-Ubych, Robert Bibik, Tomasz Latusek, Tomasz Rutkowski, Jacek Fijuth, Wojciech Fendler and Bartlomiej Tomasik

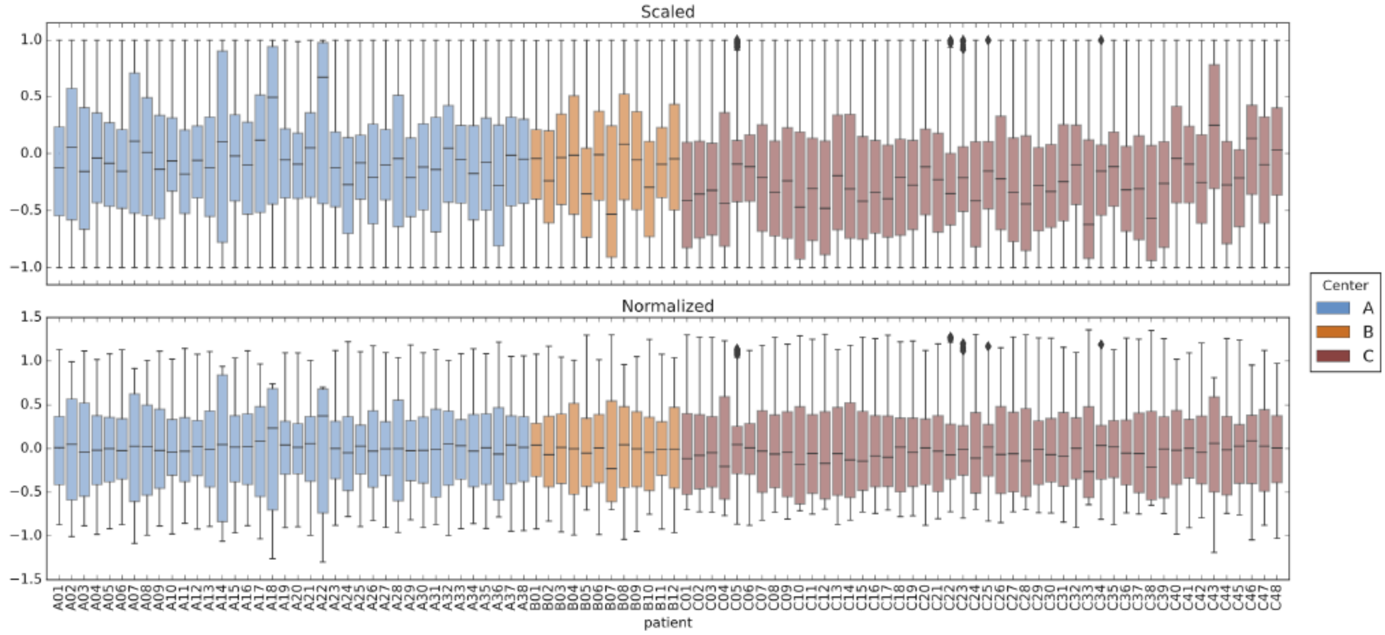

Figure S1. Scaling and normalization of radiomic features' values.

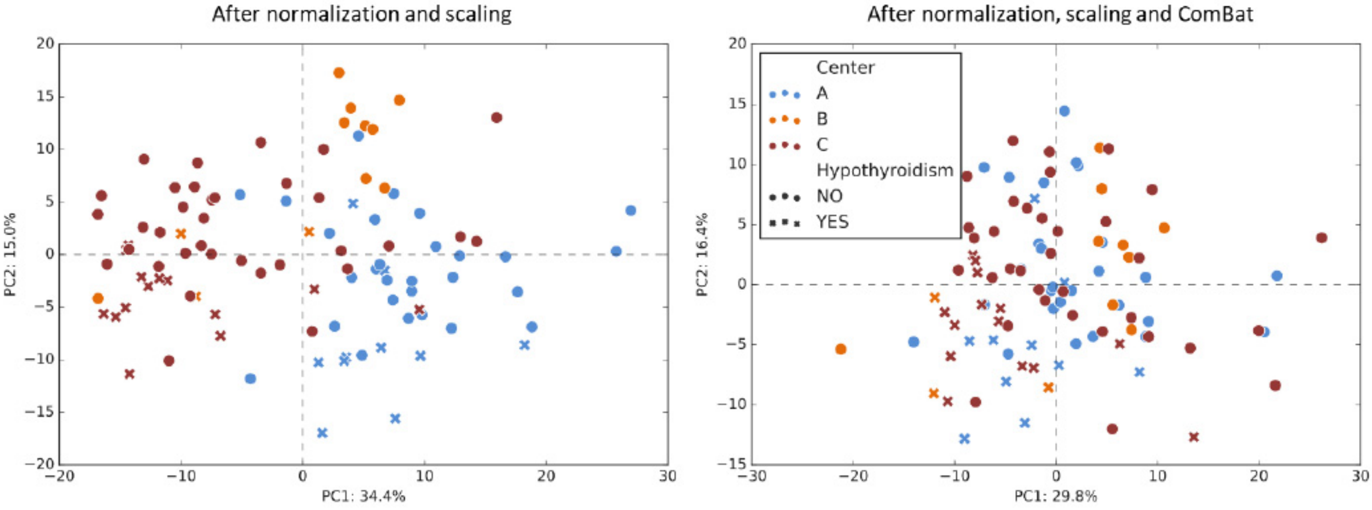

Figure S2. Principial component analysis before and after batch effect correction.

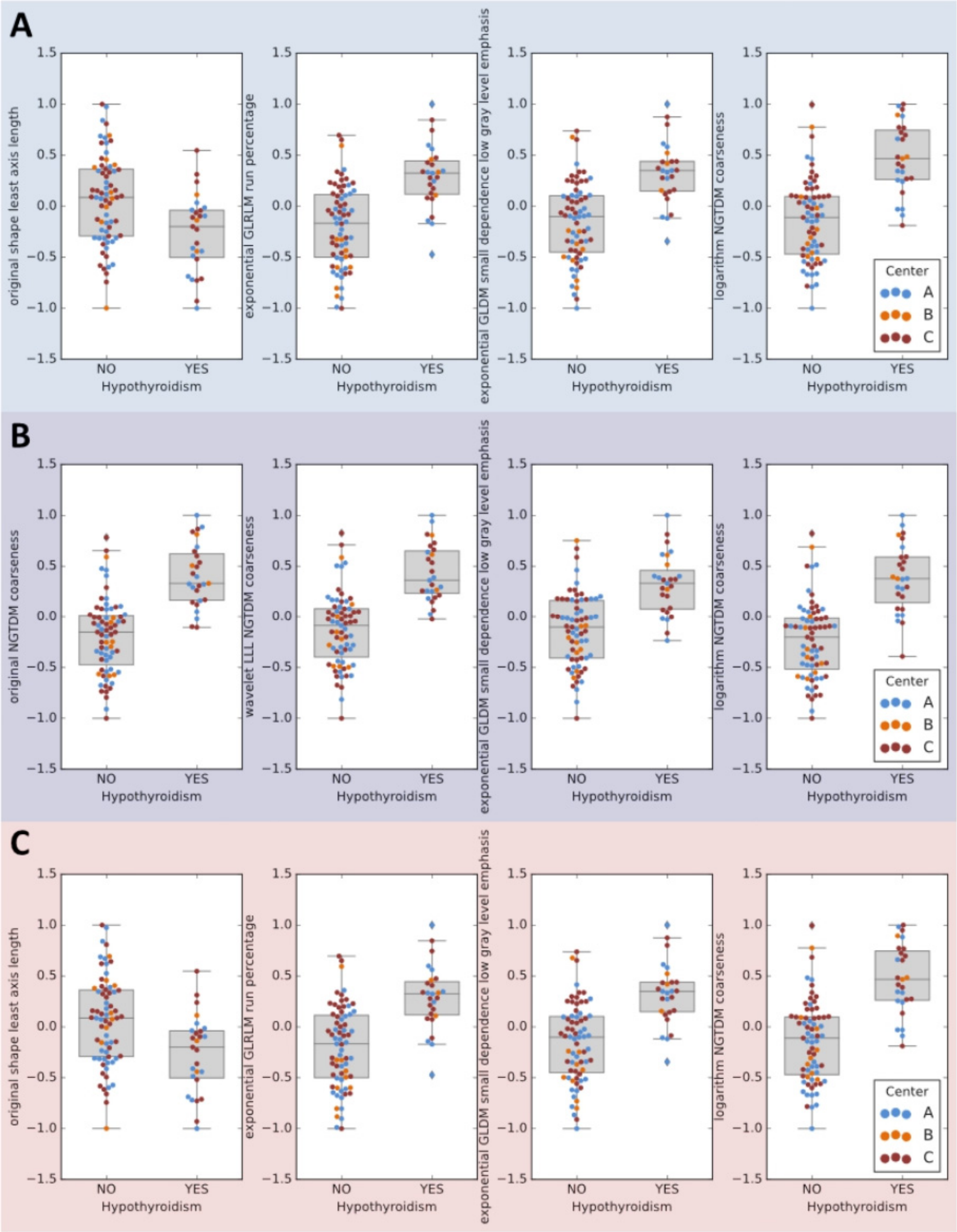

Figure S3. Transformed values of radiomic features retained in radiomic-clinical models. (**A**) variant Ia, (**B**) variant: Ib, (**C**) variant II.
